# Moving humanitarian-military relations forward: a new typology

**DOI:** 10.1186/s41018-023-00134-5

**Published:** 2023-02-24

**Authors:** Rob Grace, Maria Carinnes Alejandria, Madison Bates, Samuel T. Boland, Alexandria Nylen, Zein Tayyeb, Adam C. Levine

**Affiliations:** 1 Brown University, Providence, USA; 2 Universiti Brunei Darussalam, Bandar Seri Begawan, Brunei; 3 Chatham House, London, UK

## Abstract

This article presents a new typology for humanitarian-military relations (HMR). This typology can serve as an analytical framework for assessing, during humanitarian emergencies, how civilian responders can and should engage with armed actors. The typology considers two factors: (1) the nature of crisis-affected population’s perceptions of an armed actor, and (2) the extent of alignment of civilian responders’ and armed actors’ interests and objectives. This typology is empirically rooted in an in-depth analysis of HMR across four humanitarian response contexts: (1) the Kivu Ebola Epidemic in the Democratic Republic of the Congo, (2) the Rukban forced displacement crisis along the Jordan-Syria border, (3) the Taal volcano eruption in the Philippines, and (4) the COVID-19 pandemic in the Philippines. The analysis presented in this article is based on 175 qualitative interviews conducted with civilian responders, armed actors, and crisis-affected individuals across these contexts.

## Introduction

Three decades ago, as the twenty-first century approached, humanitarian organizations, militaries, and states collaborated in an effort to bring coherence to the field of humanitarian-military relations (HMR). In 1994, these efforts manifested in the release of the Oslo Guidelines on the Use of Foreign Military and Civil Defence Assets in Disaster Relief (Oslo Guidelines). As the years progressed, a new field emerged, dubbed by different terms across various organizations and networks: humanitarian civil-military coordination (or CMCoord) for the United Nations (UN) Office for the Coordination of Humanitarian Affairs; civil-military relations (or CMR) for the Red Cross and Red Crescent Movement; civil-military coordination (or CIMIC) for various militaries; UN CIMIC for the UN Department of Peacekeeping Operations; and humanitarian-military interaction (or HMI) for the World Food Programme. In 2003, the Oslo Guidelines were supplemented by the Guidelines on the Use of Military and Civil Defence Assets to Support United Nations Humanitarian Activities in Complex Emergencies (known as the MCDA Guidelines), which extended and adapted the Oslo Guidelines to contexts beyond disasters emanating from natural hazards. A core conceptual framework for HMR—referred to in this article as the “Three C’s” framework—was developed to conceptualize how humanitarians and militaries should engage with one another across different types of contexts (as this article later explains in greater detail). HMR stakeholders also crafted additional guidance documents focused on particular issue areas, for example, the Inter-Agency Standing Committee (IASC) Non-Binding Guidelines on the Use of Armed Escorts for Humanitarian Convoys, endorsed by the IASC in 2013. In this new era, it was hoped, engagements between humanitarian organizations and militaries in disaster response would not proceed in an ad hoc manner but rather would be systematized and guided by core principles (Metcalfe, Haysom, and Gordon 2012; Heaslip and Barber 2014).

Given all these developments, one might conclude that the field of HMR is thriving. However, humanitarian and military actors alike have lamented that existing guidance (including the Oslo and MCDA guidelines) does not actually guide decision-making in the field (Grace 2020a). Indeed, HMR practitioners lack guidance about how to approach a wide range of issues, including not only the integration of military assets into civilian response (the core issue addressed by the Oslo and MCDA guidelines) but also navigating access obstacles and promoting civilian protection with armed actors, as well as grappling with humanitarian insecurity, including the use by humanitarian agencies of armed escorts and the implementation of humanitarian notification systems (Grace 2020a; Grace and Card 2020). The vision of HMR coherence heralded by the Oslo Guidelines, and all the aforementioned subsequent developments, has given way to ad hoc approaches to grappling with the myriad challenges that emanate from civilian responders’ and armed actors’ overlapping response efforts.

This article aims to feed into efforts to reinject coherence into the field of HMR, joining an emerging strand of literature geared toward promoting empirically rooted conceptualization of how practitioners can best grapple with the key challenges of HMR practice (Rolfe 2011; Zyck 2013; Anders 2013; Heaslip and Barber 2014; Horne and Boland 2019; Bolettino and Anders 2020). Toward this end, this article proposes a new typology for understanding how civilian responders can and should engage with different types of armed actors. The authors devised this typology based on an analysis of in-depth semi-structured interviews conducted with civilian responders, armed actors, and crisis-affected populations in different types of response contexts across three geographic areas: the Democratic Republic of the Congo (DRC) (the Kivu Ebola epidemic), Syria/Jordan (the forced displacement crisis in Rukban along the Syria-Jordan border), and the Philippines (the Taal volcano eruption and the COVID-19 responses).

This article proceeds in eight parts. The first part describes in greater detail the methodology that undergirds this article. The second part presents and analyzes the logic and limitations of existing HMR guidelines, principles, and concepts. The third part proposes a new typology for HMR. The fourth, fifth, sixth, and seventh parts—empirically drawing from analysis of the aforementioned disaster response contexts in the DRC, Syria/Jordan, and the Philippines—illustrate the typology by analyzing examples of four emblematic armed actor types. The eighth part offers concluding remarks.

## Methodology

As noted, the primary empirical basis of the typology that this article will elaborate is a set of semi-structured interviews conducted with civilian responders, armed actors, and crisis-affected communities across different types of disaster response contexts. The overarching benefit of rooting this article’s analysis in the three contexts mentioned in this article’s introduction—DRC, Syria/Jordan, and the Philippines—is the breadth of disaster response context types (natural hazards, forced displacement, and large-scale disease outbreaks) and the wide range of relevant armed actor types that operated in these contexts, including militaries, non-state armed groups (NSAGs), peace operations, police, and private security contractors.

A total of 175 semi-structured interviews were conducted. Interviewees were recruited via preexisting networks from the research team, identification of key actors and stakeholders based on publicly available information, and snowball sampling. The interviews were open-ended in nature, lasting approximately one hour each. The research team used interview guides that were also iteratively elaborated on during the data collection process. Subsequently, the research team translated (when necessary) and transcribed interviews and coded interview transcripts inductively for key themes using NVivo or MAXQDA. Once all interviews had been coded, the research team produced internal memos that were then thematically compared with one another in a series of extended discussions across the research team to identify and resolve any incongruence or gaps and to avoid siloing. Table 1 (below) provides more information about the interviewee pool. The rest of this section offers additional details about each context and the conduct of research interviews.

**Table 1:** Interviewees by type and context

Interviewee type	Response context	Context-specific total	Total
Civilian responders	DRC	20	62
	Jordan	25	
	Philippines	17	
Armed actors	DRC	8	20
	Jordan	–	
	Philippines	12	
Crisis-affected community members	DRC	39	93
	Jordan	19	
	Philippines	35	
Total	DRC	67	175
	Jordan	44	
	Philippines	64	

### Democratic Republic of the Congo (Kivu Ebola epidemic)

The Kivu Ebola epidemic (2018–2020) allows for an examination of HMR in an insecure setting in which a large-scale disease outbreak intersected with a protracted armed conflict. Interviews with civilian responders captured perspectives from actors associated with 11 organizations, including three international NGOs, one international organization, five UN agencies, and one academic institution. Interviews with armed actors included UN security personnel, the UN Organization Stabilization Mission in the Democratic Republic of the Congo (MONUSCO), and the US Department of Defense. These interviews were conducted remotely since COVID-19-related travel restrictions precluded international research travel to the country. Interviewees from the Armed Forces of the Democratic Republic of the Congo (FARDC) and the Congolese National Police (PNC) are not included in the interviewee pool. Once again, COVID-19 precluded international research travel to the country, and security and risk considerations precluded local data collectors from undertaking this task. Interviews with Ebola-affected community members were conducted in person by a local partner, the Goma-based Pole Institute.

### Syria/Jordan (the forced displacement crisis in Rukban)

The forced displacement crisis in Rukban (2014–present) allows for an analysis of HMR during a geopolitically charged context where various stakeholders—including the Jordanian Armed Forces (JAF)—weighed humanitarian considerations alongside political and security interests. A key HMR issue has been international humanitarian access negotiations (during which the JAF was a key interlocutor) to reach forcibly displaced Syrians in Rukban, located in Syria just across the Syria/Jordan border. Interviews with civilian responders were conducted remotely. These interviews included actors associated with 11 humanitarian organizations (including 5 international NGOs and 6 UN humanitarian agencies) and 3 interviewees who engaged in this context for the US government. The research team sought to interview armed actors but none consented to an interview. To sample the refugee community and document their perspectives of the Rukban crisis response, the research team conducted 19 interviews with individuals who lived in Rukban before being transferred into either Zaatari or Azraq refugee camps in Jordan. Rukban itself remained inaccessible to the research team during the data gathering process.

### The Philippines (Taal volcano eruption and COVID-19)

In the Philippines, the responses to the Taal volcano eruption (2020) and COVID-19 (2020–present) represent a very different context than those in the DRC and Syria/Jordan. In this context, the key civilian responders were local government actors, local civil society actors, and local armed actors (military and police), with international civilian responders on the periphery playing a supporting role. Civilian responder interviews were conducted remotely and included five interviewees who worked in Philippine governmental roles, seven local non-governmental civilian responders, and five international humanitarian actors. Twelve uniformed personnel participated in the study, representing the following units: the Armed Forces of the Philippines (AFP); the Philippine National Police (PNP); and the Philippine Navy, Air Force, and Coast Guard. An AFP Reservist was also interviewed. Crisis-affected community interviews were conducted in person with local actors who had been affected by the Taal volcano eruption, COVID-19, or both crises.

## The logic and limitations of existing humanitarian-military relations guidelines, principles, and concepts

The plethora of existing HMR guidelines, principles, and concepts devised over the past three decades is oriented toward navigating a core overarching challenge. This problematique is how—when facing a capacity gap that armed actors can fill by engaging in direct relief or providing support in areas such as logistics, infrastructure, and/or security—civilian responders can leverage armed actors’ assets in a manner that does not compromise the civilian-led, principled nature of the response (Colona 2017). Further complicating this overarching problematique is the challenge that military actors and humanitarians hail from different professional cultures, often mistrust one another, and operate within disparate organizational structures (militaries are more hierarchical, whereas humanitarian organizations collaborate across organizational lines through more horizontal coordination structures) (Byman et al. 2000; Metcalfe, Haysom, and Gordon 2012).

Turning to the Oslo and MCDA guidelines, one can see how these documents seek to lay out principles and concepts for grappling with this overarching challenge. Both sets of guidelines emphasize the importance of three of the core humanitarian principles: humanity (addressing human suffering wherever found), neutrality (refraining from participating in hostilities or taking sides in political, religious, or ideological controversies), and impartiality (providing assistance based on need, independent of identity characteristics, prioritizing the most vulnerable). Both the Oslo and MCDA guidelines also articulate key concepts to guide civilians’ decision-making about how and when to incorporate armed actors’ assets in civilian responses. These elements include the concepts of civilian control (meaning, according to the MCDA guidelines, “While military assets will remain under military control, the operation as a whole must remain under the overall authority and control of the responsible humanitarian organization”) and last resort (as the MCDA guidelines state, “Military assets should be requested only where there is no comparable civilian alternative and only the use of military assets can meet a critical humanitarian need”) (MCDA Guidelines 2006).

**Fig. 1:**
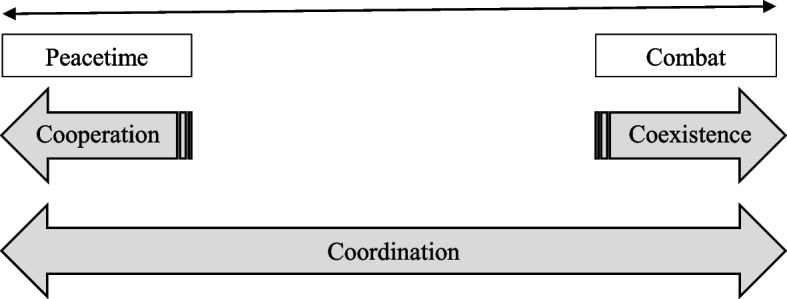
This figure is adapted from Metcalfe, Haysom, and Gordon 2012

Building on this foundation, the “Three C’s” framework conceptualizes how civilian responders should approach decisions about the depth of engagement between civilian responders and armed actors across different operational environments. The “Three C’s” framework envisions HMR across a spectrum of different types of contexts. At one end of the spectrum are responses to natural hazards during peacetime. In such environments, cooperation between civilian responders and armed actors is possible, including the potential for armed actors to engage in direct relief. At the other end of the spectrum are complex emergencies. In these conflict contexts, where civilian responders operate in areas where military combat occurs, coexistence is the proposed mode of engagement with armed actors. The notion is that, in complex emergencies, engaging with armed actors can compromise the principled nature of humanitarian response, so civilian responders should simply coexist with armed actors in the same operational environment without interacting. Figure 1 (below) presents a visual representation of this framework.

There are numerous ways in which these guidelines, principles, and concepts fall short. First, these documents do not account for the full range of armed actor types relevant to disaster response and fail to address the full scope of response context types. The Oslo and MCDA guidelines focus rather narrowly on the use of internationally deployed military assets in response operations. However, also relevant to HMR are NSAGs, police, peace operations, and private security contractors. There is the question of the applicability of the principle of last resort (relevant for international militaries) to domestic militaries, especially when a country’s military is built into a country’s disaster response coordination architecture as a first responder. Moreover, the focus on natural hazard responses versus complex emergencies glosses over, or entirely ignores, the particularities of other operational contexts, namely, large-scale disease outbreaks and contexts of forced displacement. Some research has been produced that examines HMR in certain public health emergencies and forced displacement crises, but these analyses have not yet been brought fully into the ambit of broader literature on HMR (for literature on HMR during public health emergencies, see Kamradt-Scott et al. 2016; Konyndyk 2019; Boland et al. 2020; for forced displacement see Byman et al. 2000; Terry 2001; Cook and Ne 2018; and Ahmed 2018).

Second, existing HMR guidelines, principles, and concepts also do not grapple with the full range of issues on which civilian responders and armed actors engage with one another. Incorporating armed actors’ assets into civilian responses (the primary focus of the Oslo and MCDA guidelines) and considerations about the use of armed escorts (the focus of the IASC Non-Binding Guidelines) constitute key strands of HMR but falls well short of capturing the full HMR picture. Parallel to engagements on these issues, civilian responders also engage with armed actors on issues that include negotiating humanitarian access, promoting civilian protection, and other issues of security (such as devising and implementing humanitarian notification systems). This reality points toward the importance of marrying HMR with related issue areas of humanitarian access and civilian protection, including questions about how civilian responders can maximize impact in humanitarian negotiation, humanitarian diplomacy, and confidential and public advocacy (Rolfe 2011; Grace 2020b; Clements 2020). In short, the field of HMR is more expansive and multi-dimensional than existing guidelines, principles, and concepts would leave one to believe.

Third, existing HMR guidelines, principles, and concepts are inadequately actionable. Consider the IASC Non-Binding Guidelines on the Use of Armed Escorts for Humanitarian Convoys. These non-binding guidelines frame the use of armed escorts as a practice that should be timebound and only pursued in exceptional circumstances. The operational reality contrasts sharply with this vision. In protracted armed conflicts where civilian responders face perpetual insecurity (for example, in the Sahel and Somalia), armed escorts—and an overall posture of bunkerization, by which humanitarians fortify themselves with robust security protections—has endured in perpetuity as civilian responders’ modus operandi for years (Tronc, Grace, and Nahikian 2018; Ferraro 2021).

Consider also how the “Three C’s” framework addresses complex emergencies. According to this framework, in complex emergencies, civilian responders and armed actors should simply coexist, refraining from direct engagement with one another. As the “Three C’s” spectrum moves from peaceful to conflict settings, the depth of humanitarian-military engagement should decrease, according to this framework. However, the operational reality is actually the exact opposite in many instances, when one considers the full range of ways in which civilian responders and armed actors interact. As the spectrum moves from non-conflict to conflict, the need to engage on humanitarian access and civilian protection is more likely to increase (as access difficulties become more complicated and civilian protection risks proliferate). There might be some armed actors for whom coexistence is the desired approach (for example, particularly violent NSAGs actively hostile to humanitarian actors), but the operational reality is that mere coexistence does not make sense as a blanket statement applicable to all armed actor types in conflict settings. The “Three C’s” framework—similar to the Oslo and MCDA guidelines, as well as the IASC Non-Binding Guidelines—inadequately addresses the operational realities of HMR.

Fourth, existing HMR guidelines, principles, and concepts are rooted in untested assumptions about crisis-affected community perceptions. A key concern underlying the overarching humanitarian-military problematique is that civilian responders’ engagement with armed actors could cause the crisis-affected population to perceive that the civilian response has been unduly compromised and/or instrumentalized by armed actors’ interests and aims. There has been virtually no research conducted to document the veracity of this assumption and/or to provide further clarification and nuance in understanding when and why crisis-affected populations find armed actors’ participation in civilian relief operations problematic and when and why they do not (one example of research that does address this issue is Kwaja et al. 2021). Without these empirics, one cannot know for certain whether HMR thinking is rooted in sound assumptions.

## A new typology for humanitarian-military relations

This section presents a new typology that reconceptualizes HMR and fills the gaps that the previous section highlighted. The typology is based on the intersection of two key factors. The first factor is the extent of overlap between, on the one hand, an armed actor’s organizational interests, and on the other hand, civilian responders’ views of humanitarian needs. The second factor is the extent to which the crisis-affected population views the armed actor as a credible agent of security. According to this typology, there are four emblematic armed actor types, each of which points toward a particular HMR approach from civilian responders. The authors derived this typology inductively from an analysis of the data collected for this study. This typology also validates and builds on existing literature on HMR. For example, Horne and Boland (2019) examine variations in the overlap between armed actors’ and civilian responders’ interests and aims, and Kwaja et al. (2021) probe how armed actors’ responsibility for human rights violations impacts community acceptance for HMR. Table 2 (below) lays out the typology. The rest of the section explains the two factors and then discusses four emblematic armed actor types and associated HMR approaches.

**Table 2:** A new typology for humanitarian-military relations

		**Extent of crisis-affected population’s view of armed actor as a credible agent of security**	
		**High**	**Low**
**Extent of overlap between armed actor’s and civilian responders’ interests and aims**	**High**	*Armed actor type* *Active partner* *HMR approach* *Collaborate*	*Armed actor type* *Loose cannon* *HMR approach* *Contain*
	**Low**	*Armed actor type* *Reluctant partner* *HMR approach* *Compromise*	*Armed actor type* *Disrupter* *HMR approach* *Convert*

### The extent of overlap between an armed actor’s and civilian responders’ interests and aims

One key factor in the typology that this article elaborates is the extent of overlap between an armed actors’ and civilian responders’ interests and aims. There is a range of factors that can shape the extent to which an armed actor perceives meeting humanitarian needs to be in its interest. Previous literature has analyzed a range of potentially relevant dimensions, including the need to maintain a favorable reception from the local population, the scale and length of a conflict, and the severity of politically polarized ethnic conflict in the country (Downes 2008; Labonte and Edgerton 2013; Jo 2015, Stanton 2016; Forster 2016, Grace 2020c). According to the typology, a high extent of overlap means that the armed actor’s interests and civilian responders’ objectives are generally aligned. A low extent of overlap means that the armed actors’ interests and civilian responders’ objectives are generally not aligned, and moreover, could even be directly conflictual.

### The extent of the crisis-affected population’s view of an armed actor as a credible agent of security

A second key factor in this typology is the extent of the crisis-affected population’s view of an armed actor as a credible agent of security. The typology considers two possibilities for this factor. The first possibility is that there is a high extent to which the crisis-affected community perceives that an armed actor effectively fulfills its primary security function for the benefit of the local community. The implication is that, in such circumstances, one would expect the crisis-affected community to generally welcome (or at least, refrain from objecting to) the involvement of such an armed actor in disaster response.

The second possibility is a low extent to which the crisis-affected community perceives an armed actor to be a credible agent of security. Such a perception can arise for two overarching possible reasons. The first possible reason is that the armed actor poses a direct security threat to the local population. In such an instance, the implication is that the crisis-affected community would not welcome the involvement of such an armed actor in disaster response. Indeed, it is logical to presume that, if an armed actor poses a security threat to the local community, crisis-affected actors would perceive that a disaster response that involves such an armed actor (even if the response is civilian-led) could further threaten the local community. The second possible reason is that the crisis-affected population views the armed actor not necessarily as a direct security threat but rather as negligent in its security duties. In such an instance, the local population might perceive that an armed actor’s engagement in disaster response could further distract the armed actor from its primary task of providing security.

### Four emblematic armed actor types and associated approaches for humanitarian-military relations

The two factors laid out above intersect, yielding four emblematic armed actor types, each of which is associated with a particular HMR approach from civilian responders. The first emblematic armed actor type is an active partner. For an active partner, both factors are high. The crisis-affected population views the armed actor as a credible agent of security and the armed actor’s interests and aims align with those of civilian responders. With such an armed actor, civilian responders can pursue collaboration, meaning that the armed actor (and associated assets, capabilities, and competencies) can be leveraged and incorporated into the disaster response. Since the crisis-affected population has a positive view of the armed actor, one would not expect civilian engagement with the armed actor to be objectionable. Moreover, since the armed actor’s interests and civilian responders’ objectives overlap, one would not expect the armed actor to resist participating in the response in a manner that is consistent with civilian responders’ aims. Such an armed actor presents the most likely possibility for fruitful engagement between civilian responders and armed actors in terms of HMR.

A second emblematic armed actor type is a reluctant partner. Similar to an active partner, a reluctant partner is generally viewed positively by the crisis-affected population. The difference is that the reluctant partner’s interests do not align well with civilian responders’ views of humanitarian needs. In such a case, the armed actor could very well play a fruitful role in the response but lacks incentives to do so. The HMR approach for civilian responders is compromised. The reason is that, in such a scenario, if civilian responders face capacity gaps, or other needs for which an armed actor’s cooperation is crucial (for example, facilitating humanitarian access), the armed actor is likely to be unwilling to accommodate civilian responders’ requests. Negotiation and advocacy will likely be required from civilian responders to persuade the reluctant partner to coordinate to the extent possible. However, given the underlying disconnect between the armed actor’s and civilian responders’ interests and objectives, these negotiation and advocacy efforts will likely only yield moderate results. Consequently, civilian responders will be left implementing an imperfect response in which they lack adequate resources and/or adequate cooperation from relevant armed actors in enabling access, security, and civilian protection. Hence, civilian responders will need to consider how to most effectively operate in a compromised operational environment or whether to withdraw from the context.

A third emblematic armed actor type is a loose cannon. A loose cannon is not viewed positively by the crisis-affected population but does have interests that align with civilian responders’ objectives. In this sense, a loose cannon constitutes the inverse of a reluctant partner. Whereas civilian responders need (but cannot adequately secure) sufficient coordination from a reluctant partner, a loose cannon (due to negative perceptions from the local community) can be a liability in the response but very much wishes to become involved. The HMR approach for civilian responders is containment. Such an approach can entail engaging with an armed actor in the response but doing so in a manner that reduces the visibility of the armed actor’s role.

The fourth emblematic armed actor type is a disrupter. A disrupter is the armed actor type least conducive to fruitful engagement. A disrupter is not viewed positively by the crisis-affected community and does not have interests and aims that overlap with those of civilian responders. More specifically, the local population does not view a disrupter as an agent of security, and the lack of overlap between the armed actor’s and civilian responders’ interests and objectives suggests that the armed actor does not have a stake in the crisis-affected population’s welfare, or at the very least, has priorities that diverge from those of both civilian responders and the crisis-affected population. For civilian responders engaging with disrupters, the HMR approach is to convert the disrupter into an entity that will refrain from disrupting the response. This process can entail negotiation, as well as confidential and public advocacy. Nevertheless, such efforts might still fall short, leaving civilian responders to mitigate any disruptive effects that such an armed actor might seek to inflict on a civilian response.

### Collaboration with active partners: Filipino uniformed personnel during the Taal volcano eruption response

The Taal volcano erupted in January 2020 in the Philippines, displacing over 300,000 people; disrupting critical infrastructure including transportation lines, power, and water supplies; and damaging over 14,000 homes (Al Jazeera 2021). The response was led by the civilian government, in particular, the National Disaster Risk Reduction and Management Council (NDRRMC), which operates under the Department of National Defense, and more specifically, the Office of Civil Defense, which is the Executive Arm and Secretariat of the NDRRMC (Humanitarian Advisory Group, 2020). Due to their proximity to the area, participants from the Philippine Air Force and the PNP were the first responders. Within twenty-four hours, there was a steady succession of arrival of other uniformed personnel units in the area, including the PNP, the AFP, the Philippine Coast Guard, and the Bureau of Fire and Protection. Both the AFP and the PNP played a prominent role in the response in terms of evacuation, search and rescue, managing relief supplies (including direct delivery of relief packages and loaning transportation resources for relief delivery), security and checkpoint management (including manning checkpoints to make sure people did not enter or re-enter the eruption-affected area), building temporary shelters, clearing roads of debris to facilitate transportation, promoting situational awareness (for example, the AFP, the PNP, and the Bureau of Fire and Protection were deployed to different areas to provide hourly situation reports regarding the situation on the ground), and psycho-social support (for example, the AFP’s Civil Relations Services band provided entertainment for survivors).

In this context, there has been a high overlap between armed actors’ interests and civilian responders’ views of humanitarian needs. Indeed, in the Philippines, the participation of uniformed personnel in disaster response is institutionalized and systematized. In addition to facing protracted non-international armed conflict with Maoist rebels and Islamic separatist groups (including, since 2014, NSAGs linked to Islamic State), the Philippines ranks among the world’s most vulnerable countries to natural hazards, regularly experiencing typhoons, earthquakes, large-scale floods, and landslides. Considering the country’s vulnerability to natural hazards, as well as continued political instability, the Philippines has undertaken extensive efforts to build up domestic capacity for disaster response. These efforts have included the creation of a national cluster system modeled after the UN humanitarian cluster system. This system also entails institutionalized roles for uniformed personnel, with the AFP leading the “search and rescue” cluster and the PNP leading the ‘law and order’ cluster (Humanitarian Advisory Group 2020).

Local interviewees affected by the Taal volcano eruption generally welcomed the participation of uniformed personnel in the response, perceiving that the presence of uniformed personnel promoted peace and order. For interviewees from Batangas (where the Taal volcano is located), the presence of uniformed personnel brought feelings of relief, as the military is widely perceived to be dependable during periods of emergencies. These positive perceptions of uniformed personnel persisted despite overall local criticisms of the response more generally. Crisis-affected interviewees emphasized several shortcomings, including delays in deploying rescue operations, ineffective public communication, relief supplies not aligned with needs, and poor conditions in evacuation centers (for example, excessive crowding and inadequate water and sanitation).

These two dimensions—the alignment of interests between uniformed personnel and civilian responders and the positive local view of the role of uniformed personnel in the response—enabled effective collaboration between civilian responders and uniformed personnel as active partners in the response. Findings from interviews with responders (uniformed personnel and civilian) generally aligned with those from crisis-affected interviewees. Responders generally criticized the response in general terms while pointing toward seemingly minor challenges related specifically to engagements between civilian responders and armed actors. The unanticipated scale of the eruption, and the speed at which the eruption progressed, led to a “chaotic” evacuation (as one interviewed civilian responder articulated), during which uniformed personnel were not equipped with adequate resources, resorting to using cars (as opposed to larger vehicles able to transport more people) for evacuation and personal cell phones for communication.

Nevertheless, uniformed personnel and civilian responders generally spoke positively of one another in terms of roles played in the response. For example, a member of the Philippine Air Force described civil society organizations as “a huge help” during the response and “our partner in our projects.” A civilian responder articulated a similar sentiment, drawing attention to the fact that, in this context, the identity of the responder as civilian or military/police, has been less important than the responder’s sense of duty related to the humanitarian imperative. This interviewee stated, “What is the biggest value at the end of the day is being the person, being the humanitarian person, regardless of you are… from the military, or being a big person in an organization. That doesn’t matter… At the end of the day, it’s you being a humanitarian person, understanding the whole context, and you’re doing that because you have a cause.”

To be sure, civilian responder interviewees did mention certain challenges related to engagements with uniformed personnel. One issue relates to the hierarchical nature of the military and the resulting difficulty of civilian governmental actors to coordinate with the military, given the civilians’ lack of authority, in practice, to influence or direct military actors during the response. Along similar lines, a civilian government interviewee stated that there was sometimes a need to communicate with military counterparts through an intermediary (namely, a higher-ranking military official able to wield authority) to convey messages, for example, about locations to which particular people would be evacuated or transferred. There were also instances of uniformed personnel entering the ‘danger zone’ (meaning the eruption-affected area that was unsafe for people to enter). Photographs of uniformed personnel within this area became public, leaving the impression with crisis-affected communities that it would be safe for them to return as well. A civilian governmental interviewee stated of uniformed personnel who entered the ‘danger zone’ (and posted publicly about it on social media), “Yes, we alerted them, we warned them not to go there, and… some didn’t listen.”

These challenges, however, constitute standard dimensions of managing a relationship with an active partner with whom civilian responders are collaborating. The systematized role of uniformed personnel in disaster response in the country, as well as the positive perceptions of uniformed personnel from the crisis-affected population, meant that the AFP and the PNP constituted active partners with whom civilian responders could fruitfully collaborate.

### Compromise with reluctant partners: Jordanian armed forces during the Rukban crisis

The crisis in Rukban emerged in 2014, when forcibly displaced Syrians began fleeing toward Rukan and Hadalat, located in Syrian territory within a demilitarized zone near the northeast Jordanian border. This area— an arid strip of land near the often referred to as the “Berm”—became a securitized “buffer zone” meant to prevent an ISIS resurgence in the territory (Macaron 2018). As part of this strategy, US-led coalition forces established a military base et al.-Tanf. The area around the base would later become known as the 55-km zone, given an understanding struck between the USA and Russia that the USA would retain control of a semi-circle area—which encompassed Rukban—stretching out 55 km from the base (Magruder 2020).

As forcibly displaced Syrians fled from civil-war-related violence to safety in this area, the securitized ‘buffer zone’ became a de facto humanitarian enclave. Initially, the JAF took the lead in providing relief services, including food, water, and non-food items. As the forcibly displaced population in Rukban and Hadalat continued to increase in size, the Jordanian government requested support from humanitarian organizations. During this phase, the International Committee of the Red Cross provided food, water, and medical relief, before transitioning food and water deliveries to UN agencies in early 2016. The JAF and Jordanian border security continued to support humanitarian operations as UN agencies and international NGOs scaled up. By June 2016, the estimated population of forcibly displaced people in the Berm was 77,000 (ECHO 2016).

That month, a car bombing at the “Berm” for which ISIS claimed responsibility led the Jordanian government to seal the border. After this incident, the Jordanian government severely constrained humanitarians’ ability to cross the border to the “Berm” and forcibly displaced Syrians’ ability to cross into Jordan, consequently stranding the population in the “Berm” without access to essential services. In the stark words of one humanitarian interviewee, “These people are abandoned; they have entirely nothing, nothing. No facilities for them, nothing. No rights. They’re in the middle of the desert and no humanitarian aid can access them.”

Turning now to an assessment of the JAF in terms of the two factors relevant to this article’s typology, the affected population in the “Berm” generally viewed the JAF as a credible agent of security. Crisis-affected interviewees described fleeing traumatizing events at the hands of armed actors in Syria and felt a sense of relief at seeing the Jordanian soldiers upon arrival at the “Berm.” Indeed, crisis-affected interviewees consistently characterized the Jordanian military in a positive light (for the sense of safety and security respondents felt upon being received by the JAF in the area) or a neutral light (in that interviewees expressed frustration about the lack of access to basic goods and services while not necessarily blaming the JAF specifically).

However, especially after the June 2016 car bombing, the JAF’s interests did not align well with international humanitarian organizations’ objectives. As already mentioned, whereas international humanitarian organizations sought access to the population in the ‘Berm’ (by seeking permission from Jordanian authorities to undertake cross-border operations into Syria or to transport forcibly displaced Syrians into Jordan), the JAF (as well as civilian Jordanian government personnel) severely restricted humanitarian access, driven by terrorist-linked security concerns.

In this sense, the JAF was an emblematic example of a reluctant partner. The JAF was well poised to play a role in relief efforts (given the Rukban population’s positive perception of the JAF) but lacked the willingness to do so other than in a highly controlled and limited manner.

Humanitarians in this context thus faced a grave dilemma of principles versus operational realities. Purely principled operations were not possible, but what types of compromises would be acceptable? How should humanitarians make these determinations? On the one hand, some humanitarian organizations found the environment too constrained and decided not to continue operations. One such humanitarian interviewee noted the impossibility of ensuring that humanitarian efforts would be based on needs, stating, “For us, as humanitarians, we found it unacceptable.” On the other hand, other humanitarian organizations—including UN agencies—continued efforts to serve the needs of the Rukban population, even despite the challenges encountered and compromises made.

There were three overarching approaches for humanitarian organizations that sought to maintain some degree of access to the Rukban population. First, several UN agencies, aiming to transfer relief items into the Berm without crossing the border themselves, delivered aid across the border by crane. Relief items—including food, water, and hygiene kits—were transferred over to tribal leaders, who would then deliver the aid to people in Rukban (Williams 2017). In these operations, humanitarians were unable to undertake direct distribution, and there was no way to guarantee that people in need would actually receive the aid.

Second, humanitarians seeking to undertake cross-border operations ran programming through implementing partners. However, through negotiations with the Jordanian government, these operations were only possible if humanitarians used contractors closely associated with the JAF and/or the tribal army in Syria, raising concerns about neutrality and independence (ibid).

Third, UN agencies built and ran a medical clinic that could provide emergency medical services for the most vulnerable residents in the “Berm” (UNHCR 2017). However, the process of evacuating Rukban residents to the medical clinic entailed two layers of screening controlled by armed actors. First, tribal leaders, including tribal army entities, would screen residents in the “Berm” for evacuation to the clinic. Second, the JAF would undertake medical screening before letting people access the clinic. As a result of this process, many in need of medical care in Rukban, including women with pregnancy-related complications, were not able to access the clinic.

These conditions were the best that humanitarians could negotiate, including via extensive confidential and public advocacy. In the words of one humanitarian interviewee (words echoed by other interviewees who worked for other humanitarian organizations), “My team, we did anything possible. We touched any keys. Approach by proxies, the royal family. We met the U.S. ambassador. I tried to lobby with many ambassadors: Italy, Spain. We did everything possible.” There were some lower-level humanitarian victories in the form of members of the JAF taking actions that were not officially authorized. For example, a crisis-affected interviewee discussed an episode in which a JAF physician manufactured a false pretense (asserting that a woman needed urgent medical attention) so that she could enter Jordan to reunite with family who had already crossed into the country. UN humanitarian agencies also carried out medical referrals into the Jordanian hospitals through the JAF, but only for certain cases. Additionally, humanitarian interviewees mentioned that there were instances when the JAF allowed some aid to pass through in an “off the books” manner, even if these activities had not been formally authorized. However, the scale at which these informally authorized deliveries occurred was minimal, at least compared with the scale of need in Rukban.

As this portrait of the JAF amidst the Rukban crisis illustrates, engaging with a reluctant partner inevitably entails compromise. Through negotiation, relationship-building, advocacy, and engaging with potentially influential third-party stakeholders (such as donor governments), the aim is to persuade the reluctant partner, to the extent possible, to engage with and enable the civilian response. However, given the lack of overlap between the armed actor’s interests and civilian responders’ objectives, civilian responders engaging with reluctant partners must be prepared to grapple with the realities of a context where a purely principled humanitarian response is not possible.

### Containment of loose cannons: armed escorts during the Kivu Ebola epidemic

During the Kivu Ebola epidemic (2018–2020), there were 3470 confirmed and probable cases of infection, among which 2280 people are known to have died. Amidst the response, civilian responders—including public actors, such as the World Health Organization (WHO), and international humanitarian organizations (including other UN agencies and international NGOs)—grappled with how to navigate an insecure environment. The epidemic primarily affected the North Kivu and Ituri provinces in the country’s east, where almost all households have reported at least one household member subjected to violence and/or displacement (Alberti et al. 2010). Over the course of 2018–2020, attacks in the DRC impacted 132 aid workers (including aid workers killed, wounded, and kidnapped), according to the Aid Worker Security Database. Despite these security challenges, responders eventually contained the outbreak and declared the epidemic over in June 2020.

Relevant to the security environment during the epidemic was a panoply of NSAGs, most of which are considered *Mai Mai*, a broad categorization of NSAGs in the DRC that can be loosely understood to be community defense militia. Some analysts have estimated that there are more than 100 active *Mai Mai* groups in the DRC (Morgan 2018). Many *Mai Mai* groups serve to genuinely protect communities, whereas others exploit communities through looting, cattle rustling, banditry, kidnapping for ransom, and sexual violence (UNHCR, 2013). During the Kivu Ebola epidemic, some *Mai Mai* groups contested the response and were responsible for attacks against responders, while other *Mai Mai* groups actively supported public health measures such as raising public awareness, conducting community outreach, and protecting public health infrastructure. The most notorious NSAG in the DRC (which is not considered to be a *Mai Mai* group) is the Allied Democratic Forces (ADF), a fundamentalist Islamist group that conducted an ongoing insurgency in the Ebola-affected areas.

To grapple with the security environment during the response, civilian responders sought assistance from MONUSCO (the UN peace operation in the country originally authorized approximately two decades ago), the FARDC (the national military), and the PNC (the national police). The FARDC and the PNC served as armed escorts for civilian responders and also provided site security (for example, outside Ebola Treatment Centers and national coordination office compounds). MONUSCO assisted civilian responders with site security (for example, outside hotels where international staff resided), area security (such as patrols), armed escorts (although on a smaller scale, compared with the FARDC and the PNC), risk mapping (for example, assessing which roads were safe to travel), and logistics (especially helicopter transportation, provision of vehicles on loan to the response, medevac services, provision of fuel, as well as communications and information technology support).

Each of these armed actors—the FARDC, the PNC, and MONUSCO—qualifies as a loose cannon under this article’s typology. Interviews with crisis-affected community actors revealed a low extent to which local actors perceived these armed actors to be credible agents of security. There was widespread consternation that Ebola-affected community members expressed in interviews regarding the notion that the FARDC, the PNC, and MONUSCO, in their Ebola response roles, were distracted from their principal role of mitigating insecurity and protecting civilians from the ADF. The following crisis-affected interviewee quote captures a widely held sentiment, that being that the FARDC, the PNC, and MONUSCO have failed to effectively fulfill their roles as security providers for local communities: “Nothing can be said about the army except the disappointment of people who expect more from them in terms of protection… For the police, I think the problem is the number of employees. The country should have brought more policemen here… Talking of the MONUSCO, I think it’s not even worth talking about. They are partners of the country, but we do not believe in them.” Such perceptions persisted in an environment where widespread rumors and misinformation about the response proliferated, including that the UN helped supply and/or arm the ADF; that the Ebola response and its agents are what brought Ebola to the region; that Ebola is a mythical illness or witchcraft; that Ebola is a disease deliberately brought to the country to exterminate people; or that Ebola simply was not real.

Nevertheless, MONUSCO, the FARDC, and the PNC contributed willingly to the response. For MONUSCO, interviewee comments indicated that, at lower organizational levels, MONUSCO personnel might not have been entirely enthusiastic about assuming responsibility for providing security for the response but felt bound by the chain of command to undertake these activities nevertheless. For the FARDC and the PNC, the mode by which these actors received payment from the UN for security services points toward the possibility that financial motives drove a willingness to cooperate with the response. In relation to the typical salary of a DRC soldier or policeman, the average monthly payment that response actors paid FARDC and PNC personnel for security services (claimed at $10 a day) was an enormous increase. Moreover, payments were made in cash and were poorly documented. This reality fueled local sentiments that Ebola was a lucrative “business” for responders. When asked about local perceptions of the role of armed actors in the Ebola response, one crisis-affected interviewee said of community perceptions, “They know that a soldier or policeman is always bad. They say they ate the Ebola money.” In this sense, the FARDC, the PNC, and MONUSCO were loose cannons in that all had an interest in contributing to the response but were not viewed positively by crisis-affected communities.

Civilian responders engaged in extensive debates about whether and how to rely on the FARDC, the PNC, and MONUSCO for security. Humanitarian and public health actors did not always agree on the desired approach. On the one hand, many international humanitarian responders expressed concerns about the use of armed escorts, including the potential compromise of civilian responders’ neutrality. There was a consistent view among humanitarian interviewees that the WHO did not have the operational capabilities or HMR experience to lead a public health response overlayed by a humanitarian crisis. More extensive community engagement, these interviewees conveyed, would have been a more effective means of promoting security. According to these interviewees, public health teams prioritized speed over cultural sensitivity and attention to the optics of the response, using armed escorts to force themselves into community contexts they should not have rushed into, driven by the aim of “killing the virus at all costs.” On the other hand, public health interviewees stressed the importance of science-led approaches to combating infectious diseases. These interviewees argued that humanitarians were too slow in the face of the viral spread and expressed frustration at humanitarians’ criticisms since the response ultimately “worked.”

Nevertheless, the approach toward MONUSCO was to confine MONUSCO’s engagement to back-end activities (for example, risk mapping, logistics, and low-visibility site security and area security roles). This effort to contain MONUSCO’s role evidently worked. Most Ebola-affected community interviewees reported being hardly or not at all aware of MONUSCO’s role in the response. However, civilian responders’ collaboration with the FARDC and the PNC, in light of the low local trust of government-affiliated security actors, fed into the aforementioned misinformation and rumors about nefarious motives driving the Ebola response. In this sense, this context illustrates the benefits of containing, and the detriments of failing to contain, engagement with loose cannons.

### Converting disrupters: Filipino uniformed personnel during COVID-19

In March 2020, Filipino President Rodrigo Duterte, after initially downplaying COVID-19, embraced militarized rhetoric to demonstrate a commitment to defeating the virus (Lasco, 2020). By the end of March, President Duterte had declared a state of public health emergency; a “community quarantine” for Metro Manila (the National Capital Region); and then an “enhanced community quarantine” throughout all of Luzon (where Manila is located), as well as a “State of Calamity” throughout the country (Aguilar 2020). Presidential proclamations triggered the involvement of the AFP and the PNP, which engaged in wide-ranging response activities, including implementing quarantines via checkpoint management; evacuation of stranded civilians; transportation, distribution, and supply chain support (for example, for personal protective equipment); direct medical assistance and staffing quarantine centers; and providing security for civilian response operations and for COVID-safe burials. Overall, uniformed personnel have played a very visible role in the Philippine government’s COVID-19 response. In addition to the highly visible securitized presence of uniformed personnel while managing checkpoints, ex-military officials have led response coordination via the COVID-19 National Task Force.

Filipino uniformed personnel during COVID-19—specifically, in relation to their role in implementing quarantine measures—constitute emblematic examples of disrupters. First, from the perspective of uniformed personnel, the vision of how to most effectively manage the pandemic did not align with a more health-centered approach favored by wide swaths of civilian responders. Uniformed personnel were mandated to implement a primarily “law and order” approach to contain viral spread via forcibly preventing in-country population movements, whereas many civilian responders placed greater emphasis on ensuring that, amidst the pandemic, Filipinos could retain access to medical treatment and other essential services, especially access to food and livelihoods. Many local civilian public health responders adamantly opposed the government’s overly securitized mode of managing the pandemic, publicly advocating for a shift in the government’s approach.

Second, relatedly, the militarized presence of uniformed personnel managing checkpoints alarmed many crisis-affected individuals. Over the course of 2020, reports proliferated about abuses committed by uniformed personnel during quarantine implementation, including detainee abuse related to individuals arrested at checkpoints, the shooting of a mentally ill retired soldier by policemen, and sexual and gender-based violence committed at checkpoints (Gonzales 2020; Aspinwall 2020; Human Rights Watch 2020). As one crisis-affected interviewee stated about engaging with uniformed personnel while navigating checkpoints during quarantine, reflecting a widely held view, “I was fearful because they have guns, firearms. One wrong move and you are done.” This local fear and distrust of uniformed personnel was not universal. For example, members of the military made voluntary donations that toward the response (for example, some AFP personnel donated a portion of their salary to the Office of Civil Defense), and the PNP created an “adopt-a-family” program to send relief to poverty-stricken families in Banaue, located in the province of Ifugao. These measures effectively generated good will for uniformed personnel in particular areas of the country.

Nevertheless, the prevalence of fear evoked during the government’s highly securitized quarantine implementation generally points toward uniformed personnel during COVID-19 as disrupters. Uniformed personnel enthusiastically engaged in the response in a manner that many civilian responders opposed and that evoked fear across many segments of crisis-affected communities.

As noted, civilian responders sought, via sustained advocacy toward the government, to convert uniformed personnel to engaging in the response through more health-centered approaches. These advocacy efforts were particularly fraught, given the counterterrorism context, as low-level armed conflicts persist in the country between the government and Maoist and Islamist rebel groups. The government has resorted to the widespread use of “red-tagging,” meaning the practice of labeling individuals “terrorists” within the country. Government red-tagging—the threat of which escalated when the Philippine government adopted an anti-terrorism law in July 2020—has resulted in killings, threats, harassment, arbitrary detention, and forced disappearances of human rights defenders, journalists, and other civil society actors. An interviewed local civilian responder discussed the risk of red-tagging in relation to advocating on issues related to the COVID-19 response, and in particular, efforts to push the government to expand COVID-19 testing efforts. This interviewee stated, “There is always that threat [of red-tagging], which is something we worry about… Especially since some advocacies, for example, are calling for wider testing, or what we call mass testing… Whenever I do speak out publicly that there is always that threat really that my views might be misconstrued as such.”

Some advocacy efforts on these issues have been successful. For example, in 2021, community pantries emerged as a decentralized national social movement in the Philippines oriented toward addressing food insecurity resulting from quarantine measures. Viral media attention fueled the creation of over 6000 community pantries across the country, but the government red-tagged numerous individuals associated with the community pantry movement, including Ana Patricia Non, who had begun the first pantry (Kusuma 2021). Non assertively pushed back, criticizing the government’s red-tagging practices in a press conference, as well as on social media. Ultimately, her efforts to mobilize public opinion against governmental restrictions and intimidation led to a drastic change. The PNP (which previously had inhibited and intimidated individuals running community pantries) issued a public apology and even established its own community pantries (Cabalza 2021). As this example, as well as the broader comments presented throughout this section, illustrates, efforts to convert disrupters via advocacy carry implicit risks but, at least in certain contexts, can ultimately succeed in converting an armed actor into a partner that can engage responsibly with civilian responders.

Yet, a conversion approach will not always succeed. In the Philippines during the COVID-19 response, the government did not fully embrace the more human- and health-centered approach for which civilian responders advocated. Consequently, civilian responders were left to operate within an overall response architecture that they considered to be compromised. In this sense, similar to a compromise approach with a reluctant partner, a conversion approach with a disrupter is likely to also entail an element of compromise when conversion efforts reach the limits of what can be achieved.

## Conclusion

The typology that this article has elaborated aims to present an analytical tool for assessing armed actors during HMR, informing how civilian responders can and should approach engagement. As laid out and illustrated by the empirical examples drawn from the DRC, Syria/Jordan, and the Philippines, the logic of the typology points toward collaborating with active partners, compromising with reluctant partners, containing loose cannons, and converting disrupters.

Three final observations are worthy of emphasis. First, this typology can apply to different types of armed actors (international and domestic militaries, peace operations, police, private security actors, and NSAGs) across different types of response contexts (natural hazards, conflicts, public health emergencies, forced displacement crises, and environments where one or more of these types of crises intersect). Although the emblematic examples presented in this article were limited to military, police, and peace operations, one could also assess NSAGs within the same framework. The fact that many *Mai Mai* groups contributed in various ways to the Ebola response in the DRC (although others were hostile to civilian responders) points toward the fact that NSAGs can also be potential partners in disaster response. Indeed, scholarship on the roles that NSAGs have played in pandemic response, as well as the incentives that some NSAGs have to demonstrate adherence to norms of international humanitarian law, point toward the need for further analysis of NSAGs within the field of HMR (Jo 2015; Breslawski 2022).

Relatedly, future research should examine, through the lens of this typology, HMR related to combatants who are active parties to ongoing armed conflicts. The cases examined in this article exhibit variation in this regard. The AFP is embroiled in armed conflict in the Philippines, and although two military interviewees also discussed their roles in active conflict, there was no active conflict ongoing in the areas of HMR operations that constituted the focus of analysis; MONUSCO is considered by most interview respondents to be a party to the conflict in the DRC but is nevertheless often characterizes as having (at least ostensibly) a peacemaking role; and the JAF is not considered to be a party to the armed conflict in Syria. Future research could probe the extent to which this variable shapes HMR dynamics. Cases such as Provincial Reconstruction Teams in Afghanistan and Iraq, or Russian governmental relief activities under the auspices of the Russian Ministry of Defence in Syria, are worthy of future analytical attention on this front.

Second, one should not view this typology as static but rather as a framework that can vary across geographic areas, populations, response contexts, and time. The Philippines context illustrates this reality rather starkly. Uniformed personnel constituted active partners during the Taal response but disrupters during the COVID-19 response. Moreover, even within the COVID-19 context, uniformed personnel were not uniformly feared by all segments of the population. As noted, in some areas, uniformed personnel garnered good will from local communities, especially via charitable efforts. Consider also the JAF. The Rukban case examined in this article focused primarily on the period after the June 2016 car bombing after which the Jordanian government, acting as a reluctant partner, drastically tightened border controls for forcibly displaced Syrians hoping to enter the country. In the pre-June 2016 period, however, the JAF trended more toward an active partner than a reluctant one.

Third, one can observe additional variations across different organizational levels. Within MONUSCO, personnel at lower organizational levels did not appear enthusiastic about focusing their activities on supporting public health and humanitarian responders during the Ebola outbreak, but given effective command-and-control structures, MONUSCO, as a whole, remained able to provide effective support. Conversely, humanitarian engagements with the JAF, as a reluctant partner, succeeded in prying some lower-level actors to undertake some actions that served humanitarian ends, even though these actions were not officially authorized (for example, allowed some aid to move across the border or facilitating certain Syrians’ movement into the country).

By proposing this typology, this article aims to bolster efforts to fulfill the original vision of HMR, as laid out in this article’s introduction. Much additional work is needed from policy actors, practitioners, and researchers on this front. There remains a need to push forward further on embracing the empirical realities of HMR practice and, in a responsive manner, designing guidelines, policies, and conceptual frameworks that speak directly to the experiences and challenges that crisis-affected communities, civilian responders, and armed actors face in their interactions with one another during disaster response contexts. The authors of this article hope that the typology presented here will constitute yet another brick in the pathway toward more conceptually coherent and empirically informed HMR.

## Data Availability

Not applicable.
